# SUSTAIN as a universal scoring tool for assessing sustainability development goals in African energy initiatives

**DOI:** 10.1038/s41598-025-23521-x

**Published:** 2025-11-20

**Authors:** Fotouh R. Mansour, Alaa Bedair

**Affiliations:** 1 Pharmaceutical Analytical Chemistry Department, Faculty of Pharmacy, Tanta University, Tanta, 31111 Egypt; 2 Medicinal Chemistry Department, Faculty of Pharmacy, King Salman International University, Ras Sudr, 46612 Egypt; 3 Department of Analytical Chemistry, Faculty of Pharmacy, University of Sadat City, Sadat City, 32958 Egypt

**Keywords:** Sustainability, SUSTAIN, Application, SDG, Initiatives, Energy science and technology, Engineering

## Abstract

Sustainability assessment has become an essential component of evaluating chemical methods and processes, requiring tools that deliver comprehensive and multi-dimensional evaluations. This manuscript introduces SUSTAIN, a novel and user-friendly tool designed to assess the sustainability of methods, processes and initiatives across environmental, social, and economic dimensions. Its core assessment framework is based on the 17 United Nations sustainable development goals (SDGs). By evaluating data related to key themes such as resource efficiency, energy use, emissions, waste generation, and social impact, and mapping this information onto the SDGs, SUSTAIN provides a dynamic platform to support informed decision-making for researchers, industry professionals, and policymakers. The tool uniquely evaluates the contribution to each of the United Nations’ SDGs, offering a structured analysis of their potential to advance or hinder global sustainability efforts. This paper presents the tool’s conceptual framework, including its SDG-based assessment methodology, and illustrates its practical application through real-world examples, and freely available software (bit.ly/SUSTAIN2025). To demonstrate its versatility, SUSTAIN was applied to evaluate three major energy access initiatives: Power Africa, SE4All, and climate finance initiatives. The initiatives received normalized percentage scores of 50.00, 85.29, and 82.35, respectively, reflecting their overall alignment with the SDGs on a scale from 0 to 100. This quantitative comparison highlights the tool’s utility in benchmarking sustainability performance and guiding strategic decision-making towards more sustainable development pathways.

**Supplementary Information:**

The online version contains supplementary material available at 10.1038/s41598-025-23521-x.

## Introduction

Sustainability has become an essential consideration in modern scientific and industrial practices, particularly within chemical methods and processes^1^. As the global community faces challenges such as climate change, resource depletion, and social inequalities, the need for sustainable practices has never been more urgent^2^. Chemical processes, due to their extensive use in various industries, have a profound impact on the environment, society, and economy^3^. However, assessing the sustainability of these methods is complex and requires a multidimensional approach that considers environmental, social, and economic factors^4^.

Sustainability metrics serve as standardized metrics that measure the impact of processes on these three dimensions^5^. For example, metrics such as resource efficiency, energy consumption, emissions, and waste production provide insights into the environmental impact of chemical processes^6^. Social implications, such as health and safety, employment, and economic benefits, are equally crucial in this assessment^7^. This holistic evaluation enables stakeholders to make data-driven decisions that align with sustainable development goals^8^. This manuscript introduces an innovative tool designed to assess the sustainability of methods, processes, projects and initiatives among others, highlighting its principles and application in relation to the United Nations’ SDGs. Each SDG will be discussed in detail, demonstrating how the tool evaluates the contribution of chemical processes to global sustainability efforts. To the best of our knowledge, this SDG-based holistic assessment has been not yet developed. The integration of sustainability metrics into a user-friendly tool offers a promising solution for simplifying the assessment process^9^. By collecting and analyzing data from various sources, the tool can provide real-time feedback and generate visual representations of sustainability performance^10^. This facilitates the comparison of different approaches and identifies areas for improvement, promoting the adoption of greener practices^11,12^.

## Methodology

To ensure transparency and reproducibility, the methodology for applying the SUSTAIN tool is formally defined as follows.

### Metric set and scoring rubric

The SUSTAIN framework is built upon the 17 United Nations SDGs as its core set of sustainability metrics. Each SDG is assessed independently based on the documented impacts of the process or initiative under evaluation. The scoring rubric for each SDG is a five-point ordinal scale. A score of + 2 (Strongly Fulfill) is assigned to an activity that directly and significantly advances the goal. A score of + 1 (Fulfill) indicates the activity indirectly or moderately supports the goal. A score of 0 (Neutral) means the activity has no significant positive or negative impact; this category also serves for “Not Applicable” cases. A score of -1 (Violate) is given if the activity indirectly or moderately hinders the goal. Finally, a score of -2 (Strongly Violate) is reserved for an activity that directly and significantly undermines the goal. Detailed descriptions and examples for each score per SDG are provided in Table 1, which serves as the definitive guide for scoring.

### Data sources and scoring process

The assessment is based on data extracted from literature and the official reports^13–15^. The scoring was conducted by the authors of this study. Each author independently scored the three initiatives based on the criteria in (Table 1). The scores were then compared, and any discrepancies were discussed until a consensus was reached for each SDG score for each initiative.

### Calculation of the total SUSTAIN score

Each SDG can contribute a maximum of 2 points to the total score, making the absolute maximum possible raw score 34 points for an initiative applicable to all 17 goals. If a specific SDG is deemed not applicable to the initiative or process under evaluation, it is assigned a score of 0. In this framework, not applicable SDGs are considered neutral: they neither increase nor decrease the score, but they also do not reduce the denominator. This ensures that methods, processes, and initiatives that contribute to a larger number of SDGs have more opportunity to achieve higher scores, thereby rewarding broader sustainability impact.

To ensure fairness and comparability between initiatives of different scopes, a normalized percentage score is calculated as the percentage of the total points achieved out of the maximum possible score, which is 34.

The SUSTAIN Score is calculated using the following formula:

$$\:\text{S}\text{U}\text{S}\text{T}\text{A}\text{I}\text{N}\:\text{S}\text{c}\text{o}\text{r}\text{e}\:\left(\text{\%}\right)\:=\frac{\left(\text{T}\text{o}\text{t}\text{a}\text{l}\:\text{p}\text{o}\text{i}\text{n}\text{t}\text{s}\:\text{s}\text{c}\text{o}\text{r}\text{e}\text{d}\right)}{\left(\text{M}\text{a}\text{x}\text{i}\text{m}\text{u}\text{m}\:\text{p}\text{o}\text{s}\text{s}\text{i}\text{b}\text{l}\text{e}\:\text{s}\text{c}\text{o}\text{r}\text{e}\right)}\times\:100$$

In this formula, total points scored is the sum of the scores across all 17 SDGs, with non-applicable goals contributing zero. The denominator, Maximum possible score, is set at 34 points (17 SDGs × 2 points each). This approach ensures that the final score consistently reflects performance relative to the absolute maximum, allowing for clear, reproducible, and transparent comparisons across diverse initiatives.

**Fig. 1:**
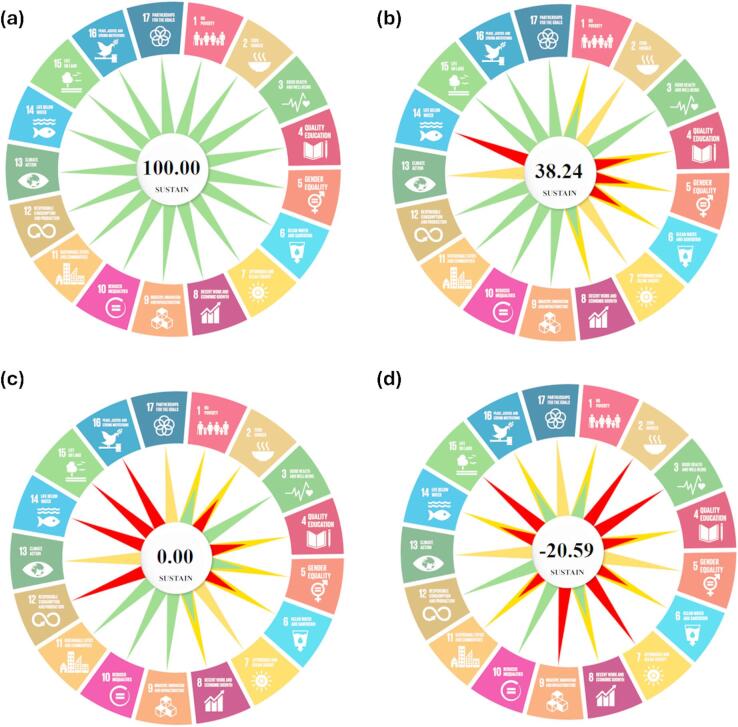
Visualization of four hypothetical sustainability performance scenarios assessed using the SUSTAIN metric. (**a**) Optimal scenario with full achievement of all 17 SDGs (all green radials); (**b**) Suboptimal yet acceptable scenario with mixed fulfillment levels (**c**) Neutral scenario with a total score of zero, indicating equal support and violation of SDGs; (**d**) Negative scenario where the initiative demonstrates a net adverse impact on SDGs.

Table 1 provides a comprehensive framework for assessing how a chemical process, product, or innovation aligns with the 17 United Nations SDGs. For each SDG, five levels of impact are defined: *Strongly Fulfill*, *Fulfill*, *Neutral*, *Violate*, and *Strongly Violate*. These categories represent the extent to which the assessed activity contributes to or detracts from the specific goal. Each category is supported by clear descriptions to aid in consistent evaluation. A numerical point system is used to quantify the assessment. If the total score is positive, the process overall supports the SDGs. A score of zero indicates a neutral impact, while a negative score suggests that the process violates or undermines the SDGs. Figure 1 presents four hypothetical scenarios illustrating varying degrees of sustainability performance based on the fulfillment of the 17 SDGs. In the first scenario (Fig. 1a), all SDGs are strongly fulfilled, representing the optimal case of maximum sustainability impact, with fully green radials indicating strong alignment with each goal. The second scenario (Fig. 1b) reflects a suboptimal or acceptable case in which some SDGs are strongly fulfilled (green radials), others are partially fulfilled (partially green radials), some are not applicable (yellow radials), and a few are either partially violated (partially red radials) or fully violated (red radials). Despite these variations, the overall SUSTAIN score remains positive, signifying that the initiative still contributes positively to sustainability. In the third scenario (Fig. 1c), the total SUSTAIN score equals zero, indicating a balance between supporting and violating the SDGs. This neutral outcome highlights areas where improvements are needed, even though some goals may still be fully achieved. Finally, the fourth scenario (Fig. 1d) illustrates a negative total score, suggesting that the initiative fails to support the SDGs meaningfully and may, in fact, hinder progress toward sustainability. This case highlights the inadequacy of current efforts and the need for significant re-evaluation and strategic redirection.

**Table 1:** Points system and examples of methods, processes, and initiatives and their impact on the different SDGs.

SDG	Category	Description	Points
SDG 1 (no poverty)	Strongly fulfill	Directly supports poverty eradication by promoting economic empowerment and improving access to essential resources.	2
	Fulfill	Indirectly contributes to poverty reduction by improving resource efficiency or creating employment opportunities.	1
	Neutral	No significant impact on poverty alleviation or economic inequality.	0
	Violate	Contributes to poverty by harming livelihoods or reducing access to resources.	-1
	Strongly violate	Severely exacerbates poverty by destroying livelihoods or causing large-scale harm.	-2
SDG 2 (zero hunger)	Strongly fulfill	Directly enhances food security and sustainable agriculture.	2
	Fulfill	Indirectly contributes to food security and sustainable agriculture.	1
	Neutral	No significant impact on food security or agricultural sustainability.	0
	Violate	Reduces food security or agricultural productivity through unsustainable practices.	-1
	Strongly violate	Causes severe harm to food systems and food security.	-2
SDG 3 (good health and well-being)	Strongly fulfill	Directly improves health outcomes, enhances sanitation, and supports disease prevention.	2
	Fulfill	Indirectly supports health and well-being by improving safety, hygiene, or environmental quality.	1
	Neutral	No significant impact on public health or well-being.	0
	Violate	Contributes to poor health outcomes through pollution or exposure to harmful chemicals.	-1
	Strongly violate	Causes severe harm to public health through illegal or unethical practices.	-2
SDG 4 (quality education)	Strongly fulfill	Directly enhances educational access, quality, and safety.	2
	Fulfill	Indirectly supports education by improving safety or access to resources.	1
	Neutral	No significant impact on education or learning environments.	0
	Violate	Negatively impacts educational outcomes through health risks or poor learning environments.	-1
	Strongly violate	Severely harms educational systems or access to learning.	-2
SDG 5 (gender equality)	Strongly fulfill	Actively promotes gender equality and addresses gender-specific health/safety needs.	2
	Fulfill	Indirectly supports gender equality through general improvements.	1
	Neutral	No direct effect on gender equality.	0
	Violate	Unintentionally contributes to gender inequality through disproportionate impacts.	-1
	Strongly violate	Actively perpetuates gender inequality or creates unsafe environments for women.	-2
SDG 6 (clean water and sanitation)	Strongly fulfill	Enhances water quality, sanitation, and sustainable management.	2
	Fulfill	Positively contributes to water and sanitation without targeted focus.	1
	Neutral	No significant positive or negative impact on water and sanitation.	0
	Violate	Unintentionally harms water quality or sanitation through pollution.	-1
	Strongly violate	Actively and severely contaminates water sources.	-2
SDG 7 (affordable and clean energy)	Strongly fulfill	Supports the development of clean, sustainable, and affordable energy.	2
	Fulfill	Contributes to energy systems but not fully renewable or clean.	1
	Neutral	No direct impact on energy sustainability.	0
	Violate	Harms energy sustainability through reliance on non-renewable resources.	-1
	Strongly violate	Undermines clean energy efforts through environmental harm.	-2
SDG 8 (decent work and economic growth)	Strongly fulfill	Promotes sustainable economic growth, job creation, and decent working conditions.	2
	Fulfill	Positively contributes to economic growth and job creation.	1
	Neutral	No significant impact on employment or economic growth.	0
	Violate	Undermines worker safety or long-term economic sustainability.	-1
	Strongly violate	Harms both worker safety and economic sustainability.	-2
SDG 9 (industry, innovation, and infrastructure)	Strongly fulfill	Promotes industrial innovation, sustainable infrastructure, and resilient processes.	2
	Fulfill	Contributes to industrial growth and infrastructure development.	1
	Neutral	No significant impact on industrialization or innovation.	0
	Violate	Hinders sustainable industrial growth and infrastructure.	-1
	Strongly violate	Actively harms industrialization and innovation.	-2
SDG 10 (reduced inequalities)	Strongly fulfill	Directly reduces inequalities by improving access to resources.	2
	Fulfill	Indirectly contributes to reducing inequalities.	1
	Neutral	No direct impact on inequality.	0
	Violate	Contributes to inequality by creating barriers or exacerbating disparities.	-1
	Strongly violate	Severely worsens inequality.	-2
SDG 11 (sustainable cities and communities)	Strongly fulfill	Directly contributes to sustainable, resilient, and inclusive urban development.	2
	Fulfill	Supports urban sustainability through efficiency or resource use.	1
	Neutral	No impact on urban sustainability.	0
	Violate	Negatively impacts urban environments.	-1
	Strongly violate	Severely harms urban sustainability and safety.	-2
SDG 12 (responsible consumption and production)	Strongly fulfill	Drives sustainable consumption and production, promoting resource efficiency.	2
	Fulfill	Contributes to sustainable production by improving efficiency.	1
	Neutral	No significant impact on responsible consumption and production.	0
	Violate	Leads to unsustainable consumption or production practices.	-1
	Strongly violate	Actively undermines responsible consumption and production.	-2
SDG 13 (climate action)	Strongly fulfill	Supports climate action through low-emission processes and carbon reduction.	2
	Fulfill	Contributes to climate mitigation or adaptation.	1
	Neutral	No direct impact on climate action.	0
	Violate	Increases greenhouse gas emissions or hinders climate mitigation.	-1
	Strongly violate	Actively worsens climate change through high emissions.	-2
SDG 14 (life below water)	Strongly fulfill	Protects marine ecosystems and prevents water pollution.	2
	Fulfill	Indirectly supports marine life by reducing pollution.	1
	Neutral	No significant impact on marine ecosystems.	0
	Violate	Harms marine life through pollution or habitat destruction.	-1
	Strongly violate	Causes severe damage to marine ecosystems.	-2
SDG 15 (life on land)	Strongly fulfill	Enhances terrestrial ecosystems and biodiversity.	2
	Fulfill	Supports terrestrial sustainability through reduced impact.	1
	Neutral	No direct impact on terrestrial ecosystems.	0
	Violate	Degrades land quality or harms biodiversity.	-1
	Strongly violate	Severely damages ecosystems and biodiversity.	-2
SDG 16 (peace, justice, and strong institutions)	Strongly fulfill	Promotes justice, safety, and ethical practices.	2
	Fulfill	Indirectly supports peace and justice through ethical practices.	1
	Neutral	No direct impact on peace, justice, or institutional strength.	0
	Violate	Contributes to environmental injustice or conflict.	-1
	Strongly violate	Actively threatens peace, justice, or security.	-2
SDG 17 (partnerships for the goals)	Strongly fulfill	Enhances global partnerships and capacity-building.	2
	Fulfill	Supports partnerships through shared resources or knowledge.	1
	Neutral	No direct impact on partnerships or capacity-building.	0
	Violate	Limits global partnerships or exacerbates disparities.	-1
	Strongly violate	Hinders international cooperation and progress.	-2

## Results and discussion

To demonstrate the practical application of the SUSTAIN tool, we evaluated three major energy access initiatives active in Africa: Power Africa, Sustainable Energy for All (SE4All), and Climate Finance Initiatives. The assessment covers the documented performance and outcomes of each initiative from its launch until the end of 2023. The scoring is based on evidence drawn from their official progress reports, independent evaluations, and data from affiliated multilateral organizations^13–15^.

### Case studies

**Fig. 2:**
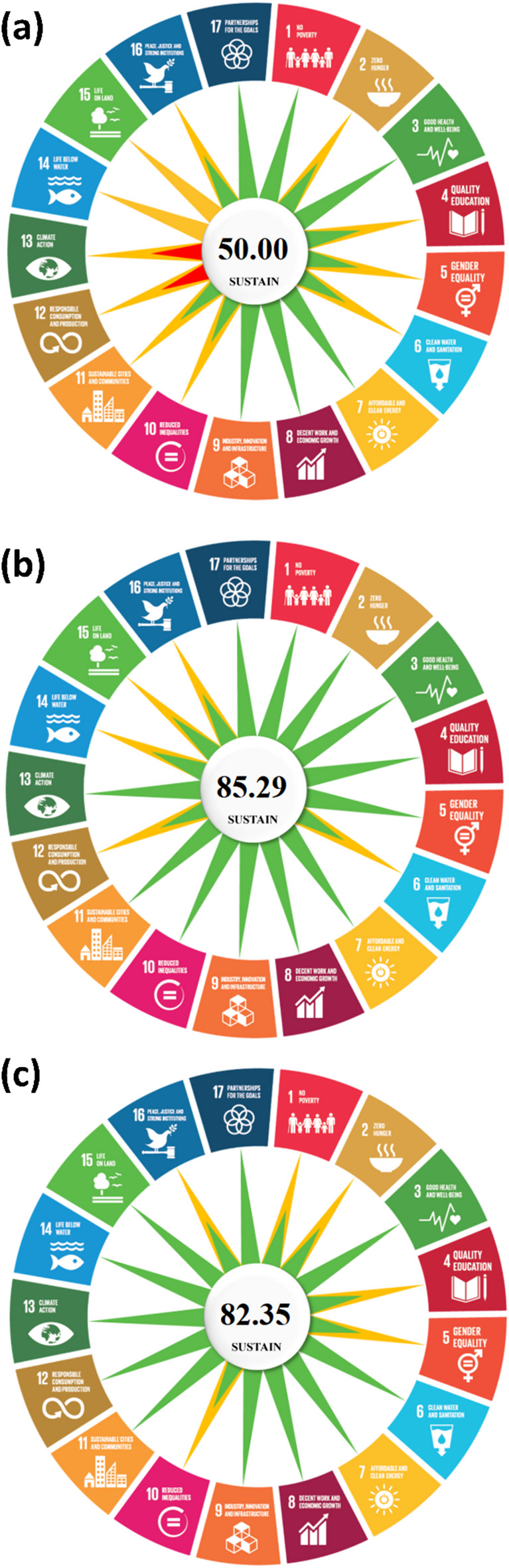
SUSTAIN scores for Power Africa (**a**), SE4All (**b**) and climate finance (**c**) initiatives.

Power Africa^13^, SE4All^14^, and Climate Finance Initiatives^15^ contribute significantly to advancing the SDGs, yet their impacts vary based on their respective approaches and priorities, as illustrated in Fig. 2. Power Africa and SE4All strongly support poverty reduction (SDG 1) by increasing energy access, with Climate Finance playing a role primarily through targeted funding mechanisms. In terms of food security (SDG 2), SE4All demonstrates the most substantial impact due to its integrated approach to energy solutions, whereas Power Africa and Climate Finance provide indirect support. All three initiatives contribute positively to health and well-being (SDG 3) by reducing indoor air pollution and improving energy availability for medical facilities. Similarly, their role in promoting quality education (SDG 4) varies, with SE4All making the most significant contributions by explicitly addressing energy needs in educational institutions, while Power Africa and Climate Finance play supportive roles.

Gender equality (SDG 5) is strongly emphasized by SE4All through targeted programs promoting women’s participation in the energy sector, whereas Power Africa and Climate Finance include gender considerations but with less explicit focus. Access to clean water and sanitation (SDG 6) benefits from all three initiatives, although Climate Finance has the most substantial impact due to its emphasis on sustainable water projects. As expected, all three initiatives strongly fulfill the goal of providing affordable and clean energy (SDG 7) and contribute to economic growth (SDG 8) by fostering job creation and industrial development. Their support for industry, innovation, and infrastructure (SDG 9) remains substantial, particularly through investments in energy-related infrastructure.

In addressing inequalities (SDG 10), SE4All demonstrates a more direct focus on equitable energy distribution, whereas Power Africa and Climate Finance provide indirect benefits. SE4All and Climate Finance strongly promote sustainable cities and communities (SDG 11), while Power Africa primarily targets rural electrification, resulting in a relatively lower impact on urban sustainability. The initiatives differ significantly in their alignment with responsible consumption and production (SDG 12). Power Africa receives a negative assessment due to its continued support for natural gas and fossil fuel projects, whereas SE4All fosters more sustainable energy consumption, and Climate Finance strongly promotes sustainability.

Climate action (SDG 13) represents another area where Power Africa’s fossil fuel investments result in a negative impact despite its contributions to renewable energy development. By contrast, SE4All and Climate Finance prioritize low-carbon energy solutions, aligning more strongly with global climate goals. Regarding biodiversity-related SDGs, Climate Finance has the strongest positive influence on life below water (SDG 14) and life on land (SDG 15) due to its focus on sustainable environmental projects. SE4All demonstrates limited but positive contributions, while Power Africa remains largely neutral in these domains.

Finally, all three initiatives contribute to peace, justice, and strong institutions (SDG 16) by promoting governance and institutional capacity-building. They also play a crucial role in fostering international partnerships for sustainable development (SDG 17), with Power Africa, SE4All, and Climate Finance all relying on cross-sector collaborations and global partnerships to advance their objectives. Based on these findings, it can be concluded that each initiative contributes meaningfully to the SDGs. Moreover, Power Africa’s engagement with fossil fuel projects presents limitations in fully aligning with sustainability principles, particularly in climate action and responsible consumption.

### Novelty and comparison with existing assessment tools

Several robust frameworks exist for sustainability assessment, each with distinct strengths. Life Cycle Assessment (LCA) and Multi-Criteria Decision Analysis (MCDA) are powerful, data-intensive methods that provide deep, granular insights into environmental impacts or complex trade-offs, respectively^16,17^. Similarly, Triple Bottom Line (TBL) indices effectively aggregate environmental, social, and economic performance into a standardized format^18^. Furthermore, numerous organizations have developed frameworks for mapping projects to the SDGs, often qualitatively describing synergies and trade-offs.

The SUSTAIN tool builds upon these foundations but introduces key innovations that address the following specific gaps. Unlike many SDG-mapping frameworks that remain qualitative or descriptive, SUSTAIN provides a standardized, quantitative scoring system (-2 to + 2) for each of the 17 SDGs. This transforms the SDGs from a communicative framework into an analytical metric, allowing for direct comparison between diverse initiatives. While LCA is exceptionally detailed, it can be resource-intensive and often focuses predominantly on environmental parameters. SUSTAIN is designed to incorporate social and economic dimensions with the same structural weight as environmental ones, using the SDG framework. It offers a more holistic view than a pure LCA and is less data-intensive, making it suitable for preliminary screening, policy analysis, and comparing initiatives where full-scale LCA is not feasible. Unlike static TBL indices or complex MCDA outputs, SUSTAIN’s output includes a dynamic radial diagram (Fig. 1) that provides an immediate, intuitive visual snapshot of a project’s sustainability profile across all 17 goals. This visual synthesis is a key feature for communicating complex assessment results to policymakers, stakeholders, and non-experts. The tool is not limited to a specific sector (e.g., only chemistry or only energy). Its structure, based on the universally accepted SDGs, allows for the comparative assessment of a wide range of initiatives, from industrial processes to large-scale development programs, as demonstrated in the case studies. It is crucial to distinguish between “greenness,” which focuses predominantly on environmental impacts (e.g., pollution, resource use), and “sustainability,” which is a broader, multi-dimensional concept encompassing social, economic, and environmental pillars, as embodied by the 17 SDGs. Consequently, established metrics of greenness, such as modified green start area (MoGSA)^19^, modified green analytical procedure index (MoGAPI)^20,21^, analytical green star area (AGSA)^22^, and carbon foot print reduction index (CaFRI)^23^, green environmental assessment and rating for solvents (GEARS)^24^ are inherently limited; they cannot be used to assess the different social and economic SDGs, as they lack the necessary scope. SUSTAIN is designed to fill this gap.

### Sensitivity analysis

The assignment of specific weights to each SDG is a critical step in tailoring the SUSTAIN assessment to reflect particular priorities. To test the robustness of the comparative conclusions derived from this weighted framework, a sensitivity analysis was performed using alternative policy-focused weighting scenarios. Three distinct scenarios were constructed, each representing a different strategic priority. An Environmental Weighting scenario assigned double the original weight to SDGs 6, 7, 13, 14, and 15. A Social Weighting scenario applied this same doubling to SDGs 1, 2, 3, 4, 5, and 10. Finally, an Economic/Industrial Weighting scenario doubled the weights for SDGs 8, 9, and 12.

The results of this analysis, detailed in Supplementary Table S1, demonstrate a high degree of robustness in the SUSTAIN metric’s outcomes while also identifying a key point of sensitivity. The relative performance ranking of the initiatives proved robust under both the Environmental and Social weighting scenarios, with the ordinal ranking of SE4All first, followed by Climate Finance Initiatives and then Power Africa remaining unchanged from the default weighting. However, under the Economic/Industrial Weighting scenario, the Climate Finance Initiatives achieved a normalized score of 85.00, drawing equal with SE4All. This shift creates a tie for the top rank and is directly attributable to the high performance of Climate Finance on the up-weighted goals, particularly SDG 12 (Responsible Consumption and Production), where it scored + 4 after weighting, compared to Power Africa’s score of -2.

This result enhances the SUSTAIN’s utility as a diagnostic tool. It reveals that the distinction between the top-performing initiatives is most sensitive to priorities surrounding economic and industrial goals. For stakeholders who prioritize SDGs 8, 9, and 12, the Climate Finance Initiatives represent a sustainability performance on par with SE4All. Conversely, the finding that Power Africa consistently ranked third across all scenarios robustly identifies it as the lower-performing initiative of the three, regardless of the chosen policy priority. This deep understanding of both stability and sensitivity is a key strength of the SUSTAIN assessment.

### Limitations

While the SUSTAIN framework provides a valuable tool for standardized sustainability assessment, several limitations should be acknowledged. The quality of the SUSTAIN assessment is directly dependent on the availability and quality of the underlying data used for the evaluation. In cases where project data is incomplete, outdated, or non-transparent, the accuracy of the score may be compromised. The tool performs best when applied to initiatives with comprehensive and publicly available reporting. The case studies presented focus on large-scale, multi-national energy initiatives in Africa. The tool’s applicability to smaller-scale projects, different sectors, or other geographical contexts, while theoretically sound, has not been empirically demonstrated in this work and remains an area for future research. These limitations, however, also present clear pathways for further development and refinement of the SUSTAIN methodology.

## Conclusion

This study introduced the Sustainability Universal Scoring and Tracking Application (SUSTAIN), a novel and user-friendly tool designed to provide a quantitative, holistic assessment of sustainability aligned with the United Nations’ Sustainable Development Goals. The SUSTAIN framework transforms the qualitative SDG framework into a standardized scoring system, enabling the direct comparison of diverse initiatives across environmental, social, and economic dimensions. The tool as available as a user-friendly application at bit.ly/SUSTAIN2025.The application of SUSTAIN to three major energy initiatives in Africa—Power Africa, SE4All, and Climate Finance Initiatives—successfully demonstrated its utility, yielding distinct scores of 50.00, 85.29, and 82.35, respectively. These results not only provide a clear, comparative benchmark for these programs but also validate SUSTAIN ‘s capacity to identify specific strengths and weaknesses in sustainability performance, such as the trade-offs between energy access and climate action observed in the Power Africa case. Despite its promising applications, the SUSTAIN tool has certain limitations. The tool’s effectiveness is dependent on the availability and quality of transparent data from the assessed initiatives. Looking forward, several pathways for future research and tool enhancement are apparent. First, applying SUSTAIN to a wider array of initiatives, including smaller-scale projects and different sectors such as agriculture, manufacturing, and waste management, would further validate its universal applicability. Second, developing a user-friendly offline software would facilitate SUSTAIN applicability. By providing a standardized and intuitive platform for SDG-based assessment, SUSTAIN offers a critical step forward in enabling more transparent, comparable, and data-driven decisions for a sustainable future.

## Supplementary Information

Below is the link to the electronic supplementary material.

Supplementary Material 1

## Data Availability

Data will be made available upon reasonable request.
