# Supplementary material for: SUSTAIN as a universal scoring tool for assessing sustainability development goals in African energy initiatives
Source: Sci Rep. 2025 Nov 20;15:40920. doi: 10.1038/s41598-025-23521-x (PMC12635146; doi:10.1038/s41598-025-23521-x)
Supplement: Supplementary file 1 — Supplementary Material 1 [file 41598_2025_23521_MOESM1_ESM.docx]

**SUSTAIN as A Universal Scoring Tool for Assessing Sustainability Development Goals in African Energy Initiatives**

*Fotouh R. Mansour^1,2*^, Alaa Bedair^3^*

**Table S1:** Sensitivty analysis of the defailut weighting

|  |  | **Default (No Weighting)** | | | **Environmental Weighting** | | | **Social Weighting** | | | **Economic/Industrial Weighting** | | |
| --- | --- | --- | --- | --- | --- | --- | --- | --- | --- | --- | --- | --- | --- |
| **SDG** | **Goal** | **Power Africa** | **SE4All** | **Climate Finance** | **Power Africa** | **SE4All** | **Climate Finance** | **Power Africa** | **SE4All** | **Climate Finance** | **Power Africa** | **SE4All** | **Climate Finance** |
| **SDG 1** | No Poverty | 2 | 2 | 1 | 2 | 2 | 1 | 4 | 4 | 2 | 2 | 2 | 1 |
| **SDG 2** | Zero Hunger | 1 | 2 | 1 | 1 | 2 | 1 | 2 | 4 | 2 | 1 | 2 | 1 |
| **SDG 3** | Good Health and Well-being | 2 | 2 | 2 | 2 | 2 | 2 | 4 | 4 | 4 | 2 | 2 | 2 |
| **SDG 4** | Quality Education | 1 | 2 | 1 | 1 | 2 | 1 | 2 | 4 | 2 | 1 | 2 | 1 |
| **SDG 5** | Gender Equality | 1 | 2 | 1 | 1 | 2 | 1 | 2 | 4 | 2 | 1 | 2 | 1 |
| **SDG 6** | Clean Water and Sanitation | 1 | 1 | 2 | 2 | 2 | 4 | 1 | 1 | 2 | 1 | 1 | 2 |
| **SDG 7** | Affordable and Clean Energy | 2 | 2 | 2 | 4 | 4 | 4 | 2 | 2 | 2 | 2 | 2 | 2 |
| **SDG 8** | Decent Work and Economic Growth | 2 | 2 | 2 | 2 | 2 | 2 | 2 | 2 | 2 | 4 | 4 | 4 |
| **SDG 9** | Industry, Innovation and Infrastructure | 2 | 2 | 2 | 2 | 2 | 2 | 2 | 2 | 2 | 4 | 4 | 4 |
| **SDG 10** | Reduced Inequalities | 1 | 2 | 1 | 1 | 2 | 1 | 2 | 4 | 2 | 1 | 2 | 1 |
| **SDG 11** | Sustainable Cities and Communities | 1 | 2 | 2 | 1 | 2 | 2 | 1 | 2 | 2 | 1 | 2 | 2 |
| **SDG 12** | Responsible Consumption and Production | -1 | 1 | 2 | -1 | 1 | 2 | -1 | 1 | 2 | -2 | 2 | 4 |
| **SDG 13** | Climate Action | -1 | 2 | 2 | -2 | 4 | 4 | -1 | 2 | 2 | -1 | 2 | 2 |
| **SDG 14** | Life Below Water | 0 | 1 | 2 | 0 | 2 | 4 | 0 | 1 | 2 | 0 | 1 | 2 |
| **SDG 15** | Life on Land | 0 | 1 | 2 | 0 | 2 | 4 | 0 | 1 | 2 | 0 | 1 | 2 |
| **SDG 16** | Peace, Justice and Strong Institutions | 1 | 1 | 1 | 1 | 1 | 1 | 1 | 1 | 1 | 1 | 1 | 1 |
| **SDG 17** | Partnerships for the Goals | 2 | 2 | 2 | 2 | 2 | 2 | 2 | 2 | 2 | 2 | 2 | 2 |
| SUSTAIN Score | | 50.00 | 85.29 | 82.35 | 43.18 | 81.82 | 86.36 | 54.35 | 89.13 | 76.09 | 50.00 | 85.00 | 85.00 |
